# Thromboembolic events and vascular dementia in patients with atrial fibrillation and low apparent stroke risk

**DOI:** 10.1038/s41591-024-03049-9

**Published:** 2024-06-05

**Authors:** Alastair R. Mobley, Anuradhaa Subramanian, Asgher Champsi, Xiaoxia Wang, Puja Myles, Paul McGreavy, Karina V. Bunting, David Shukla, Krishnarajah Nirantharakumar, Dipak Kotecha

**Affiliations:** 1 Institute of Cardiovascular Sciences, University of Birmingham, Birmingham, UK; 2 NIHR Birmingham Biomedical Research Centre, University Hospitals Birmingham NHS Foundation Trust, Birmingham, UK; 3 West Midlands NHS Secure Data Environment, University Hospitals Birmingham NHS Foundation Trust, Birmingham, UK; 4 Institute of Applied Health Research, University of Birmingham, Birmingham, UK; 5 Clinical Practice Research Datalink, Medicines and Healthcare products Regulatory Agency, London, UK; 6 Patient and Public Involvement Team, Birmingham, UK; 7 Primary Care Clinical Research, NIHR Clinical Research Network West Midlands, Birmingham, UK

**Keywords:** Atrial fibrillation, Stroke, Dementia

## Abstract

The prevention of thromboembolism in atrial fibrillation (AF) is typically restricted to patients with specific risk factors and ignores outcomes such as vascular dementia. This population-based cohort study used electronic healthcare records from 5,199,994 primary care patients (UK; 2005–2020). A total of 290,525 (5.6%) had a diagnosis of AF and were aged 40–75 years, of which 36,340 had no history of stroke, a low perceived risk of stroke based on clinical risk factors and no oral anticoagulant prescription. Matching was performed for age, sex and region to 117,298 controls without AF. During 5 years median follow-up (831,005 person-years), incident stroke occurred in 3.8% with AF versus 1.5% control (adjusted hazard ratio (HR) 2.06, 95% confidence interval (CI) 1.91–2.21; *P* < 0.001), arterial thromboembolism 0.3% versus 0.1% (HR 2.39, 95% CI 1.83–3.11; *P* < 0.001), and all-cause mortality 8.9% versus 5.0% (HR 1.44, 95% CI 1.38–1.50; *P* < 0.001). AF was associated with all-cause dementia (HR 1.17, 95% CI 1.04–1.32; *P* = 0.010), driven by vascular dementia (HR 1.68, 95% CI 1.33–2.12; *P* < 0.001) rather than Alzheimer’s disease (HR 0.85, 95% CI 0.70–1.03; *P* = 0.09). Death and thromboembolic outcomes, including vascular dementia, are substantially increased in patients with AF despite a lack of conventional stroke risk factors.

## Abstract (web summary)

Atrial fibrillation is associated with an increased risk of thromboembolic events and vascular dementia in patients with no previous history of stroke or any conventional risk factors, compared to patients without atrial fibrillation.

## Main

AF is the most common cardiac rhythm disorder with over 60 million cases expected worldwide by 2050 (ref. ^1^). Prevalence has continued to rise, doubling every few decades as a result of improved survival from cardiovascular disorders, an aging population and better screening technology^2,3^. This poses a major public health concern as AF leads to a poor patient quality of life, excess morbidity and mortality, and a large financial burden on global health systems. AF is known to substantially increase thromboembolic risk, with one in four patients admitted with a stroke having AF^4^, and strokes secondary to AF leading to greater neurological damage^5^.

Oral anticoagulation in patients with AF reduces the incidence of thromboembolism and mortality^6^; however, thromboembolic risk is not homogenous and anticoagulant use must be balanced with the risk of major bleeding. Numerous clinical tools are available to estimate stroke risk in AF, but these have only a modest predictive capacity. In a meta-analysis of eight studies, the median area under the receiver operator curve was only 0.600 for CHA_2_DS_2_-VASc (consisting of category-based points for congestive heart failure, hypertension, age, diabetes, previous stroke or thromboembolism, vascular disease and female sex^7^). Risk scores are typically based on historical observational studies that do not reflect the current burden of risk factors or their management, and do not consider other thromboembolic-related outcomes such as vascular dementia^8,9^. Guidelines from the UK National Institute for Health and Care Excellence (NICE)^10^, European Society of Cardiology (ESC)^11^ and others recommend anticoagulant use only in patients with an elevated stroke risk score. This may be too late to prevent cognitive decline^12^, and ongoing randomized trials in lower risk populations will take time to complete^13^.

This study used population data from primary care, representing current risk factor burdens from contemporary clinical practice. The objective was to evaluate whether AF was associated with a broader range of thromboembolic outcomes and sequalae despite a low perceived risk of stroke, including vascular dementia and mortality.

## Results

**Fig. 1:**
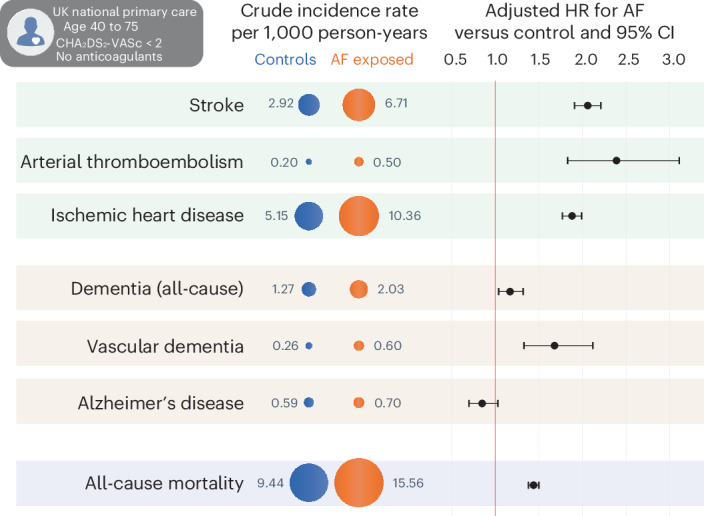
Clinical outcomes in patients with AF compared to matched controls. Comparison of clinical outcomes between patients with a diagnosis of AF and controls, matched by age, sex and health authority region. Circles denote crude incidence rate of outcomes per 1,000 person-years. For each outcome, the HR point estimate (black circle) and 95% CIs (error bars) are presented, adjusted for age, sex, Townsend deprivation score, ethnicity, hypertension and diabetes mellitus. The sample sizes used to derive statistics for each outcome are presented in Tables 2 and 3.

**Extended Data Fig. 1:**
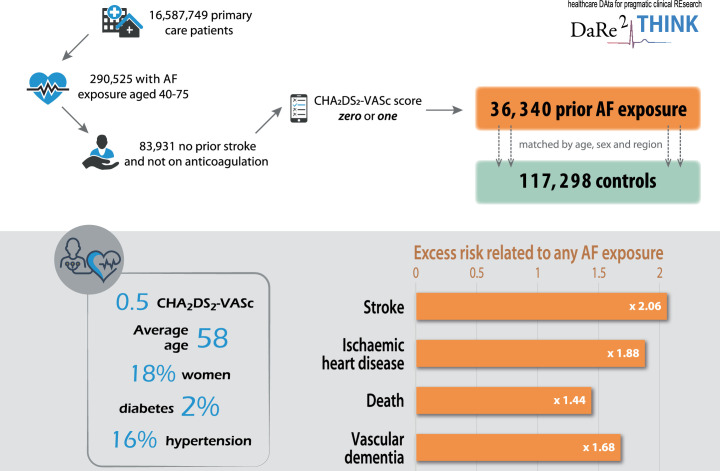
Graphical abstract. Graphical abstract summarizing the patient population, baseline characteristics and key results. AF = Atrial fibrillation.

**Extended Data Fig. 2:**
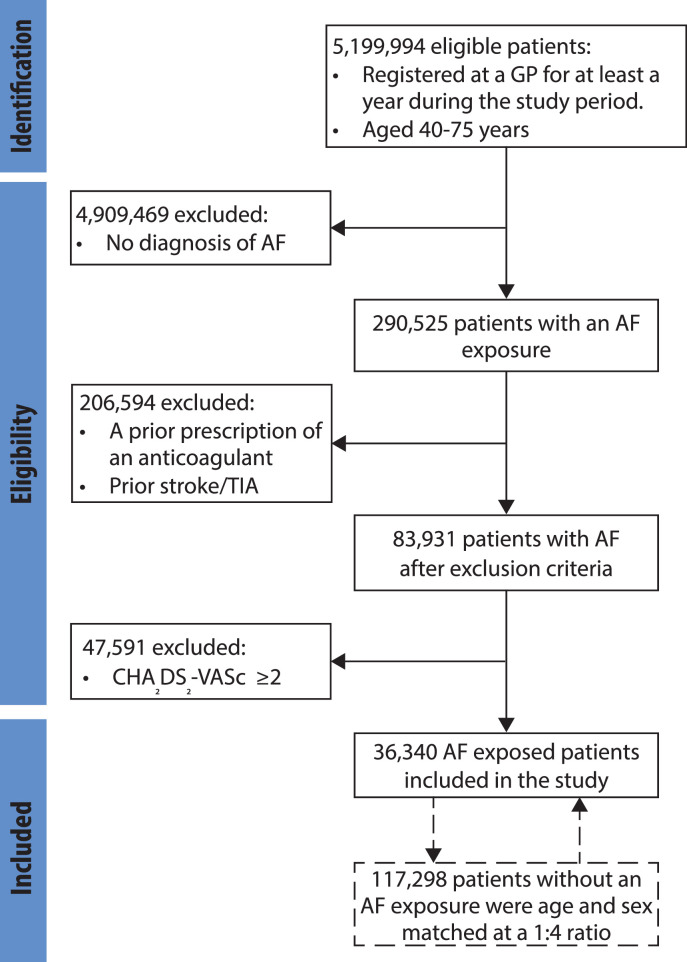
Study Flowchart. Flowchart describing the number of patients included in the study and the reasons for patent exclusions. AF = atrial fibrillation; GP = general practice; TIA = transient ischemic attack.

Data from 828 eligible general practices were used in this study, including a total of 16,587,749 patients. There were 5,199,994 eligible patients aged 40–75 years within the study period registered with an eligible practice for at least 1 year, of which 290,525 had an AF diagnosis (5.6%); Extended Data Fig. 1. After exclusion of those with previous stroke, in receipt of anticoagulants or CHA_2_DS_2_-VASc score ≥2, the study population included 36,340 patients with an AF diagnosis. These were age, sex and region-matched to 117,298 without an AF diagnosis (Extended Data Fig. 2). Demographics were comparable in the AF and control groups (Table 1), with overall median age of 58.2 years (interquartile range (IQR) 51.2–63.1), 18.0% women, 2.1% with diabetes and 16.5% with hypertension. These were consistent with a population at low predicted risk of stroke according to current risk stratification schemes; mean CHA_2_DS_2_-VASc score 0.5 (s.d. 0.5) and CHA_2_DS_2_-VA score 0.3 (s.d. 0.5). The median follow-up period was 4.4 years for those with AF (IQR 1.9–8.0) and 5.0 years for controls (IQR 2.2–8.7), with a combined total of 831,005 person-years of follow-up for outcomes (Fig. 1).

**Table 1:** Baseline demographics by AF diagnosis

Characteristic at baseline	AF diagnosis	Matched controls
	*n* = 36,340	*n* = 117,298
Age at index, years		
Mean (s.d.)	58.5 (8.9)	56.7 (8.4)
Median (IQR)	59.5 (52.5; 64.5)	57.8 (50.8; 62.7)
Age categories, *n* (%)		
<55 years	11,825 (32.5%)	45,253 (38.6%)
55–69 years	20,599 (56.7%)	65,504 (55.8%)
≥70 years	3,916 (10.8%)	6,541 (5.6%)
Sex, *n* (%)		
Women	6,560 (18.0%)	20,205 (17.2%)
Men	29,780 (83.0%)	97,093 (82.8%)
Ethnicity, *n* (%)		
White	17,616 (48.5%)	49,626 (42.3%)
Black	130 (0.4%)	989 (0.8%)
South Asian	208 (0.6%)	1,486 (1.3%)
Mixed	64 (0.2%)	235 (0.2%)
Other	227 (0.6%)	1,004 (0.9%)
Missing	18,095 (49.8%)	63,958 (54.5%)
Socioeconomic status, *n* (%)^a^		
Townsend 1 (least deprived)	8,836 (24.3%)	27,794 (23.7%)
Townsend 5 (most deprived)	3,274 (9.0%)	11,434 (9.8%)
Previous diabetes, *n* (%)	764 (2.1%)	2,747 (2.3%)
Previous hypertension, n (%)	5,982 (16.5%)	13,079 (11.2%)
Previous stroke, *n* (%)	0 (0%)	0 (0%)
Previous thromboembolism, *n* (%)	645 (1.8%)	748 (0.6%)
Previous myocardial infarction, *n* (%)	657 (1.8%)	1,156 (1.0%)
Previous ischemic heart disease, *n* (%)	3,178 (8.8%)	4,076 (3.5%)

^a^13.7% had missing information on socioeconomic status.

### Vascular and thromboembolic events

**Fig. 2:**
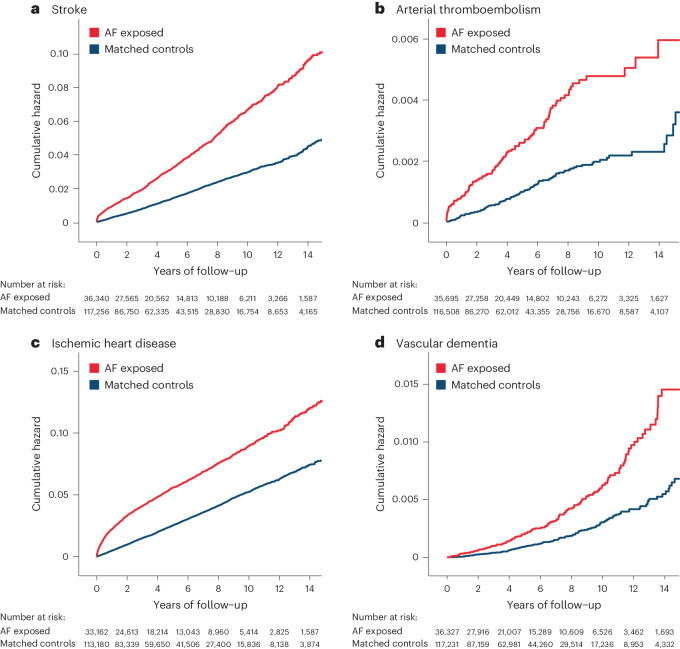
Kaplan–Meier plots for thromboembolic outcomes. **a**–**d**, Unadjusted Kaplan–Meier failure curves comparing patients with AF and matched controls for outcomes with contributing thromboembolic mechanisms, including incident stroke (**a**), incident arterial thromboembolism (**b**), incident ischemic heart disease (**c**) and incident vascular dementia (**d**). See Extended Data Fig. 3 for venous thromboembolism and Extended Data Fig. 4 for other dementia outcomes.

AF was associated with a twofold increase in vascular and thromboembolic events (Table 2). Incident stroke occurred in 1,366 (3.8%) patients with AF compared to 1,796 (1.5%) in the matched control group during a total of 818,317 person-years of follow-up (Fig. 2a). The adjusted HR of stroke for AF versus control was 2.06 (95% CI 1.91–2.21; *P* < 0.001). Arterial thromboembolic events occurred in 104 (0.3%) patients with AF versus 123 (0.1%) in controls during 829,951 person-years of follow-up (Fig. 2b), with adjusted HR 2.39 (95% 1.83–3.11; *P* < 0.001). Ischemic heart disease was recorded in 1,870 (5.6%) patients with AF compared to 3,030 (2.7%) in controls during 768,717 person-years of follow-up (Fig. 2c), with adjusted HR 1.88 (95% CI 1.77–1.99; *P* < 0.001).

**Table 2:** Vascular and thromboembolic outcomes

Outcome	AF exposed	Matched controls	AF versus control, adjusted HR (95% CI)^a^
**Stroke**			2.06 (1.91–2.21) *P* < 0.001
Events/number of patients, *n* (%)	1,366/36,340 (3.8%)	1,796/117,298 (1.5%)	
Person-years of follow-up	203,451	614,866	
Crude incidence rate/1,000 person-years	6.71	2.92	
**Arterial thromboembolism**			2.39 (1.83–3.11) *P* < 0.001
Events/number of patients, *n* (%)	104/36,340 (0.3%)	123/117,298 (0.1%)	
Person-years of follow-up	208,1230	621,829	
Crude incidence rate/1,000 person-years	0.50	0.20	
**Ischemic heart disease**			1.88 (1.77–1.99) *P* < 0.001
Events/number of patients, *n* (%)	1,870/33,162 (5.6%)	3,030/113,222 (2.7%)	
Person-years of follow-up	180,521	588,195	
Crude incidence rate/1,000 person-years	10.36	5.15	
**Pulmonary embolism**			1.23 (1.08–1.39) *P* = 0.001
Events/number of patients, *n* (%)	365/36,047 (1.0%)	808/117,063 (0.7%)	
Person-years of follow-up	205,965	618,617	
Crude incidence rate/1,000 person-years	1.77	1.31	
**Deep vein thrombosis**			0.97 (0.86–1.10) *P* = 0.620
Events/number of patients, *n* (%)	345/35,982 (1.0%)	981/116,790 (0.8%)	
Person-years of follow-up	205,576	615,858	
Crude incidence rate/1,000 person-years	1.68	1.59	

^a^Using Cox proportional hazards regression models for each outcome, adjusted for age, sex, Townsend deprivation index, ethnicity, previous hypertension and diabetes mellitus, with a two-tailed *P* value.

**Extended Data Fig. 3:**
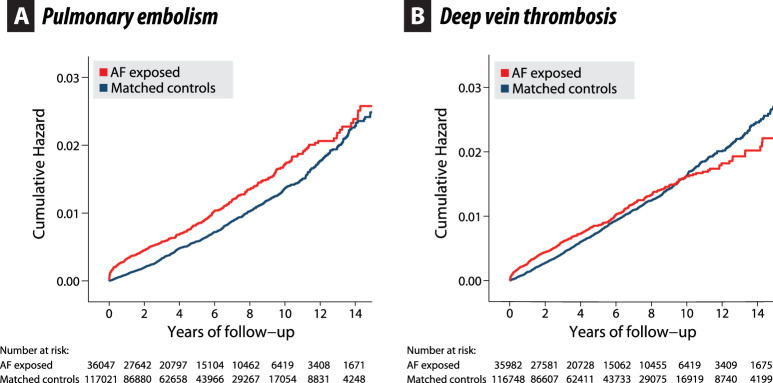
Venous thromboembolism. Unadjusted Kaplan–Meier failure curves comparing patients with atrial fibrillation (AF) and matched controls for (**a**) incident pulmonary embolism and (**b**) incident deep vein thrombosis.

The incidence of pulmonary embolism was significantly higher in those who had AF compared to controls (adjusted HR 1.23, 95% CI 1.08–1.39; *P* = 0.001; 824,582 person-years of follow-up) although Kaplan–Meier curves converged over time (Extended Data Fig. 3). There was no difference between AF and controls for deep vein thrombosis (adjusted HR 0.97, 95% CI 0.86–1.10; *P* = 0.62; 821,434 person-years of follow-up).

### All-cause mortality

**Fig. 3:**
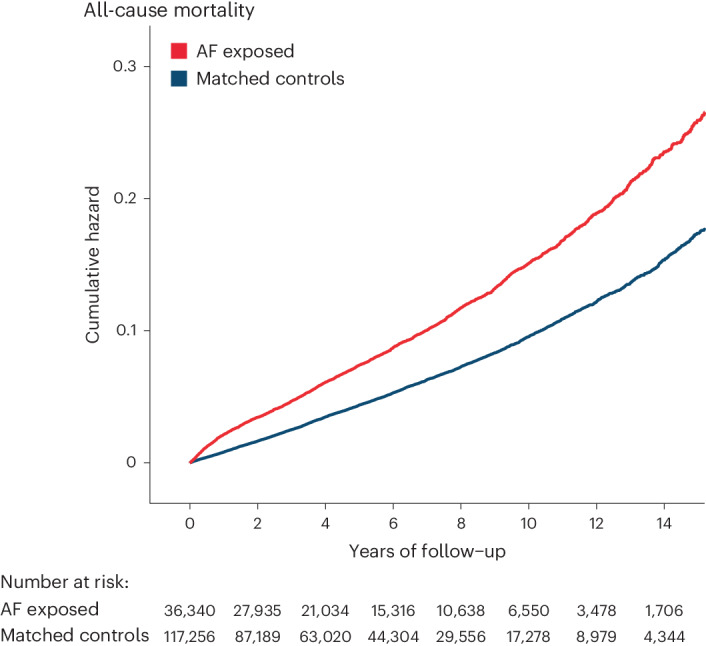
Kaplan–Meier plot for all-cause mortality. Unadjusted Kaplan–Meier failure curves comparing patients with AF and matched controls for all-cause mortality.

AF was associated with a significantly higher risk of all-cause mortality when compared to controls (Table 3 and Fig. 3); 3,246 (8.9%) patients with AF died compared to 5,875 (5.0%) in the matched control group during a total of 831,005 person-years of follow-up. The adjusted HR for all-cause mortality for AF versus control was 1.44 (95% CI 1.38–1.50; *P* < 0.001).

**Table 3:** Mortality and dementia outcomes

Outcome	AF exposed	Matched controls	AF versus control, adjusted HR (95% CI)^a^
**All-cause mortality**			1.44 (1.38–1.50) *P* < 0.001
Events/number of patients (%)	3,246/36,340 (8.9%)	5,875/117,298 (5.0%)	
Person-years of follow-up	208,611	622,394	
Crude incidence rate/1,000 person-years	15.56	9.44	
**Dementia (all-cause)**			1.17 (1.04–1.32) *P* = 0.010
Events/number of patients, *n* (%)	420/36,232 (1.2%)	788/117,056 (0.7%)	
Person-years of follow-up	207,274	619,696	
Crude incidence rate/1,000 person-years	2.03	1.27	
**Vascular dementia**			1.68 (1.33–2.12) *P* < 0.001
Events/number of patients, *n* (%)	126/36,327 (0.4%)	162/117,273 (0.1%)	
Person-years of follow-up	208,287	621,897	
Crude incidence rate/1,000 person-years	0.60	0.26	
**Alzheimer’s disease**			0.85 (0.70–1.03) *P* = 0.090
Events/number of patients, *n* (%)	145/36,304 (0.4%)	364/117,208 (0.3%)	
Person-years of follow-up	208,145	621,230	
Crude incidence rate/1,000 person-years	0.70	0.59	

^a^Using Cox proportional hazards regression models for each outcome, adjusted for age, sex, Townsend deprivation index, ethnicity, previous hypertension and diabetes mellitus, with a two-tailed *P* value.

### Dementia outcomes

**Extended Data Fig. 4:**
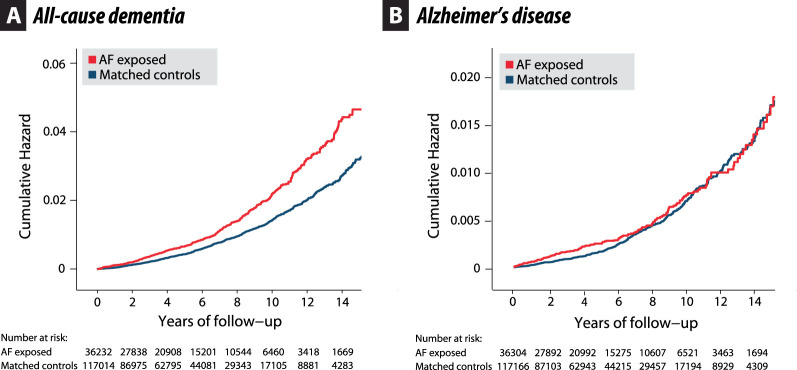
All-cause dementia and Alzheimer’s dementia. Unadjusted Kaplan–Meier failure curves comparing patients with atrial fibrillation (AF) and matched controls for (**a**) all-cause dementia and (**b**) Alzheimer’s disease. For vascular dementia, see main paper Fig. 2d.

A dementia diagnosis was recorded in 420 (1.2%) patients who had AF, compared to 788 (0.7%) patients without AF during 826,970 person-years of follow-up, with adjusted HR 1.17 (95% CI 1.04–1.32; *P* = 0.010); Table 3 and Extended Data Fig. 4a. This was driven by the association between AF diagnosis and the development of vascular dementia; 126 (0.4%) in patients with AF versus 162 (0.1%) in controls during a total of 830,185 person-years of follow-up. The higher incidence of vascular dementia associated with AF was comparable to the thromboembolic outcomes (Fig. 2d), with adjusted HR 1.68 (95% CI 1.33–2.12; *P* < 0.001). There was no association between AF diagnosis and Alzheimer’s disease (adjusted HR 0.85, 95% CI 0.70–1.03; *P* = 0.09; 829,376 person-years of follow-up); Table 3 and Extended Data Fig. 4b.

### Subgroup and sensitivity analyses

**Extended Data Fig. 5:**
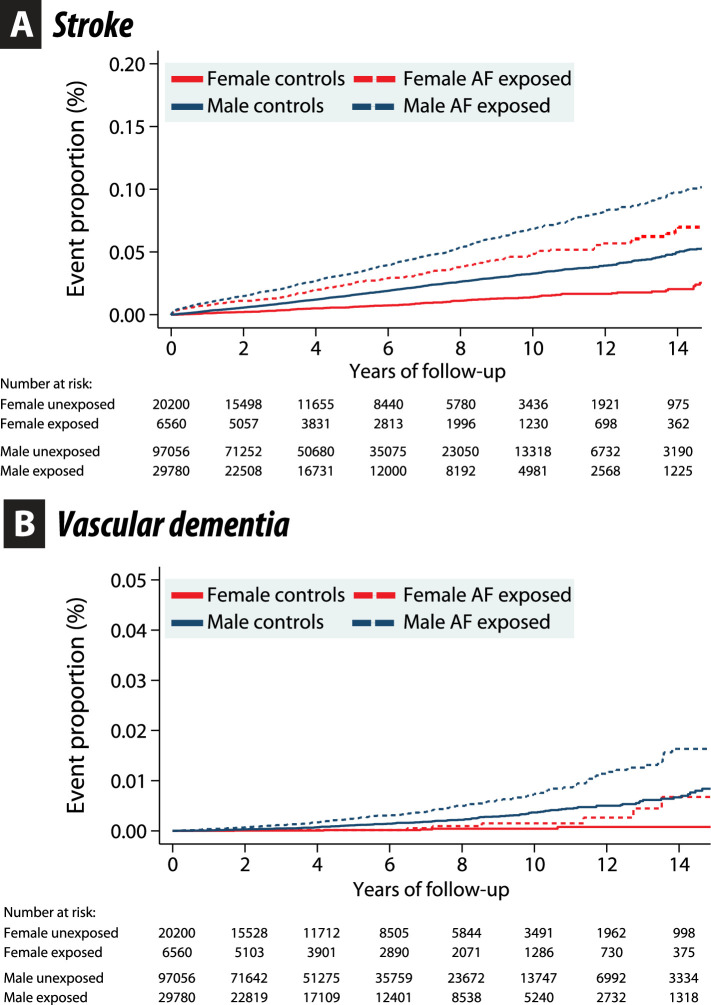
Gender difference in stroke and vascular dementia events. Unadjusted Kaplan–Meier curves comparing male and female, with atrial fibrillation (AF) and unexposed controls for (**A**) stroke and (**B**) vascular dementia.

There were no substantive differences in results for any of the prespecified subgroup analyses (Extended Data Table 1). Isolating those with new AF only, the HRs for key outcomes were similar to those for the whole population, apart from arterial thromboembolism where there were insufficient event numbers. Interaction *P* values for age as a continuous variable were nonsignificant for vascular dementia (*P* = 0.795) and all-cause mortality (*P* = 0.058). The HRs for the other outcomes including stroke, arterial thromboembolism and ischemic heart disease decreased with age (interaction *P* values 0.011, 0.004 and 0.007, respectively) among patients with AF compared to patients without AF. Although women had lower event rates, Kaplan–Meier plots indicated a similar impact of AF versus no AF in women and men for incident stroke and incident vascular dementia (Extended Data Fig. 5). Censoring at the time of anticoagulant prescription had no effect on the results. We did not observe any significant competing risk from death for stroke and vascular dementia events, or from ischemic stroke and ischemic heart disease for vascular dementia.

## Discussion

With access to nationwide, real-world healthcare data, this study demonstrates that AF was still associated with an increased risk of adverse thromboembolic outcomes despite a low perceived risk of stroke. The rates of incident stroke, ischemic heart disease and arterial thromboembolism were twice that seen in patients without AF. The association of AF with a higher rate of incident vascular dementia was consistent with the thromboembolic adverse outcomes, and we saw no correlation with Alzheimer’s disease. This suggests a thromboembolic mechanism contributing to vascular dementia in the context of AF and highlights another sequalae that clinicians should be aware of and attempt to prevent. Even after adjustment for confounding factors, all-cause mortality was 44% higher in those with AF, a facet of this condition that rarely receives clinical attention in younger patients or those perceived as being at ‘low’ risk.

The link between AF and thromboembolic events is well established, but studies have typically included older patients with multiple comorbidities or high stroke risk using current risk factor scoring tools^14^. This study extends that evidence to younger patients and those with a low burden of risk factors that would normally not be considered for prevention of thromboembolism in routine clinical practice. The associations between AF, cognitive decline and dementia are also well described, although past studies have been unable to account for underlying vascular disease (including from risk factors such as hypertension and diabetes that also portend a higher risk of AF) or the confounding use of oral anticoagulation^15–18^. The ability to separate out etiology of dementia is also important, as identified in this study where Alzheimer’s disease was unrelated to an AF diagnosis. In contrast, vascular dementia is a biologically plausible end point for AF, with multi-faceted contribution from clinical and silent embolic stroke, cerebral hypoperfusion and particularly, vascular risk factors. We demonstrated that the association between AF and vascular dementia was independent of incident ischemic stroke and ischemic heart disease; however, that does not imply independent causation, as there are multiple and shared underlying contributors. Progressive cerebral damage in patients with AF has previously been shown to be highly age-dependent^19^, but this study and others suggest this risk should be considered far earlier in the management approach to AF. In a pooled analysis of three studies with 175 patients undergoing AF ablation at a median age of 60 years, 14 (8%) had evidence of a silent infarction on brain imaging before the procedure, and white matter hyperintensity lesions were seen in 81 patients (46%)^20^. Cerebral lesions rapidly progress over time; SWISS-AF studied 1,737 patients with AF, mean age 73 years and no history of stroke, and demonstrated large noncortical or cortical infarcts in 387 (22%), small noncortical infarcts in 368 (21%) and white matter lesions in 1,715 (99%) patients^9^.

A dedicated focus on prevention of cognitive impairment should start early after the diagnosis of AF, as evidenced by a randomized trial of 973 patients with AF aged 75 years or older, where no difference in Mini-Mental State Examination at 33-months was seen comparing warfarin to aspirin^12^. Based on the neuroimaging studies discussed, this was likely too late in the pathological process for anticoagulation to help prevent further cerebral damage, supported by observational data on the duration of anticoagulation^21^. Prevention of cognitive decline and dementia requires a multi-faceted approach that also considers the contribution of comorbidities in this patient group, as well as changes in risk factors over time. A more dynamic approach to assessment of thromboembolic risk is warranted in routine clinical practice, and clinical trial data have shown the potential for integrated management of patients with AF to reduce hospital admission^22^. Further trials are needed to understand if these approaches can also lower the risk of adverse cognitive outcomes.

The annual event rates for stroke or arterial thromboembolism (0.7%) and death (1.6%) demonstrated in the current study for patients with AF at low perceived risk are consistent with other population data, and could indicate the need for earlier consideration of risk modification^23^. Although observational studies have been published in recent years showing less cognitive decline or dementia in patients with AF treated with anticoagulants^24,25^, the use of anticoagulants for this purpose should not be rationalized based on non-randomized data. Prescription biases are present in relation to the use of anticoagulants^26^ and a meta-analysis of research in the field includes ten studies with the lowest mean CHA_2_DS_2_-VASc score of 2.1 (ref. ^27^). This study did not analyze anticoagulant treatment effects as it would be inappropriate to do so with the profound biases present in routine clinical practice. Contrary to other drug therapies (for example, more digoxin use in patients at higher risk of adverse prognosis)^28^, there is reverse causation in the case of anticoagulants. Patients who have dementia, or are at highest risk of developing dementia, are less likely to receive these drugs due to concern from clinicians about bleeding and/or treatment efficacy.

At present, there are no published randomized trials that have assessed the value of anticoagulation in patients at low perceived risk of thromboembolism. There are two trials ongoing that are investigating the earlier use of a direct oral anticoagulant (DOAC) to prevent dementia; DaRe2THINK (NCT04700826) and BRAIN-AF (NCT02387229). DaRe2THINK is randomizing patients to either starting any DOAC or continuing standard of care. Using a data-driven healthcare-embedded approach for long-term follow-up^13^, this trial will test whether earlier use of DOACs is effective and cost-effective at preventing thromboembolic events (primary outcome) and cognitive decline (key secondary outcome) in patients with AF aged 55–73 years and a CHA_2_DS_2_-VASc score <2. The BRAIN-AF trial randomizes AF patients aged 30–62 years at low risk of stroke who currently do not have an indication for anticoagulation to receive either 15 mg rivaroxaban or standard of care, with key outcomes of thromboembolic events and cognitive decline. These trials will be critical to understanding whether there is value to commencing DOAC therapy at an earlier stage of the AF lifecycle, especially as the annualized event rate for a formal diagnosis of vascular dementia in these relatively young age groups is <0.1%.

This community cohort study was able to include a large sample size of patients from a broad and representative^29^ population of primary care practices with a long follow-up period; however, despite these advantages, incident events such as vascular dementia accrue at a low rate, which limits further examination. This type of study design also has several limitations that must be considered. Outcomes were derived directly from real-world clinical care, although the accuracy and completeness of medical coding in the United Kingdom is known to be high^30,31^. This study includes those with a known diagnosis of AF based on healthcare coding, including past or resolved AF. It is likely that had we been able to select only those patients with current AF or ongoing symptoms, the relationship with adverse outcomes even in this young population may have been even stronger. Conversely, patients with resolved AF are known to have ongoing risk of thromboembolism^32^, particularly as AF clinically or on an electrocardiogram is merely a manifestation of underlying atrial disease and systemic thrombo-inflammation. Thromboembolic risk and comorbidities were assessed at each patient’s baseline entry point, and risk profiling was not repeated over time. Furthermore, due to the nature of retrospective cohort studies we cannot establish causality and can only show association. Although we report the correlation between AF and vascular dementia in this study, this does not imply that AF directly caused dementia and we are unable to control for other confounders that link AF with vascular dementia.

In summary, patients with AF who are deemed to have low chance of thromboembolism using conventional risk factor scoring are still at a substantially increased risk of incident stroke, arterial thromboembolic events, vascular dementia and death compared to matched controls. Randomized controlled trials are needed and ongoing to determine whether these patients could benefit from earlier use of oral anticoagulants to improve prognosis and prevent cognitive decline.

## Methods

### Study design and data source

This is a population-based, retrospective, matched, open cohort study conducted using real-world healthcare data collected between 1 January 2005 and 31 December 2020 from the IQVIA Medical Research Database (IMRD), a proprietary database of Cegedim (France). IMRD is a UK primary care database containing pseudonymized medical records of patients registered with general practices across the UK using the VISION clinical system^33^. The UK National Health Service (NHS) is free at the point of delivery to all citizens, with general practice making up 90% of healthcare usage. It is only possible to be registered with one primary care provider, and any healthcare utilization outside of this setting, for example a hospital admission, is subsequently shared with this provider and then coded on their electronic healthcare record. Multiple studies have shown concordance between primary and secondary NHS care^30,34,35^. IMRD comprises over 18 million patient records from 832 general practices in the UK. The database contains information on patient demographics, coded records of diagnoses and symptoms using the read code clinical classification system, dispensed prescriptions, and additional health information such as physical and biochemical measurements. High data quality is incentivized through the Quality and Outcomes Framework^36^. Data extraction for this study was conducted using the Data Extraction for Epidemiological Research (DExtER) tool^37^.

### Ethics

Data collection for IMRD was approved by the NHS South-East Multicentre Research Ethics Committee in 2003. Under the terms of this approval, each study protocol undergoes independent review from the Scientific Review Committee (approval July 2017; reference no. SRC 17THIN061).

### Study population

Practices were considered eligible 1 year after the establishment of the VISION clinical system within their practice or 1 year after reporting mortality rates comparable to national averages^38^, whichever was the latest. Adults aged between 40 and 75 were considered for inclusion into the study, registered during the study period with an eligible general practice for at least 1 year.

### Exposure

Patients with a recorded diagnosis of AF were included in the exposed cohort. For patients with a new diagnosis of AF after their eligibility, the index date was assigned as the date of AF diagnosis. For patients with an AF diagnosis before their eligibility, the index date was assigned as the date of patient eligibility. For each exposed patient, up to four patients without an AF diagnosis were randomly selected after matching for age (±1 year), sex and the health authority region of the patient’s registered general practice. The same index date of the exposed patient was assigned to the corresponding matched unexposed patients to avoid immortal time bias^39^.

### Exclusions

The following exclusions were applied to exposed and unexposed cohorts: (1) patients with a record of stroke before the index date; (2) patients with a recorded prescription for an anticoagulant before the index date, either vitamin K antagonists or DOACs; and (3) patients with a CHA_2_DS_2_-VASc score ≥2 (two or more of the following one-point conditions: heart failure; hypertension; age 65 years or older; diabetes mellitus; previous myocardial infarction, peripheral artery disease or aortic plaque; and female sex).

### Covariates

Age, sex, socioeconomic deprivation, ethnicity, diabetes and hypertension were considered as confounders. Age was modeled as a continuous variable. Socioeconomic deprivation was recorded as the Townsend deprivation index categorized into quintiles^40^. Ethnicity was categorized into (1) white; (2) Black Afro-Caribbean; (3) South Asian; (4) mixed or multiple ethnicities; and (5) other ethnic group. Patients with missing Townsend and ethnicity data were aggregated into a separate missing category within that variable. The temporal pattern of AF (paroxysmal, persistent or permanent) was not considered as these classifications do not correspond to underlying pathology, are poorly recorded, physician-dependent and not relevant for consideration of stroke prevention therapy^11^.

### Follow-up and outcomes

Outcomes of interest were all-cause mortality, incident stroke, thromboembolism, deep vein thrombosis, pulmonary embolism, ischemic heart disease, dementia (all-cause), vascular dementia and Alzheimer’s disease. Patients were followed up from the index date until the earliest of the following time points: (1) recording of the outcome of interest; (2) patient censorship due to death or de-registration from their registered practice; (3) practice censorship due to ceasing of their data contribution to IMRD; and (4) study end date of 31 December 2020. Bleeding outcomes were not evaluated as these are primarily related to anticoagulation use in patients with AF (excluded at baseline in this study) and are known to be a rare occurrence when considering patients at such low estimated risk of thromboembolism^23^.

### Statistical analysis

Baseline characteristics of the exposed and the unexposed cohort were summarized and tabulated. Continuous variables were assessed using mean and s.d. or median and IQR, depending on the normality of distribution. Categorical and binary variables were summarized using numbers and percentages. Incidence rates for each of the outcomes were calculated by dividing the number of incident diagnoses by the total person-years of follow-up of at-risk patients. Patients with that outcome recorded before the index date were excluded. Cox proportional hazards regression models were generated for each outcome with associated 95% CI. Adjusted models accounted for age, sex, Townsend quintile, ethnicity, previous hypertension and diabetes mellitus. The proportional hazards assumption in Cox models was tested using Schoenfeld residuals. Subgroup analyses were conducted for key outcomes (stroke, arterial thromboembolism, ischemic heart disease, vascular dementia and all-cause mortality) for three stratifications by age (<55, 55 to 69 and ≥70 years). Two sensitivity analysis were conducted: (1) patients with new AF only along with their matched unexposed patients; and (2) censoring at the time of initiating treatment with an anticoagulant. Interactions were assessed in the fully adjusted Cox model, and a competing risk analysis for stroke and vascular dementia accounting for death was conducted using the method of Fine and Gray. Post hoc analyses were: (1) stratification of incident stroke and incident vascular dementia according to sex; and (2) competing risk analysis for incident vascular dementia accounting for incident stroke and incident ischemic heart disease. All analysis were conducted in Stata (StataCorp), with a two-tailed *P* value <0.05 denoting statistical significance.

### Reporting frameworks

The results of this study are reported according to RECORD (REporting of studies Conducted using Observational Routinely-collected health Data); see supplementary checklist.

This study meets all five of the CODE-EHR minimum framework standards for the use of structured healthcare data in clinical research^41,42^, with three out of five standards meeting preferred criteria; see supplementary checklist.

- CODE-EHR Framework Domain 1: dataset construction and linkage
  - Source of dataset: study data were sourced from IMRD. IMRD is a UK primary care database containing pseudonymized medical records of patients registered with general practices across the UK using the VISION clinical system. Data extraction and transformation was performed using the DExtER tool^37^.
  - Approach to missing data: IMRD uses real-world data collected directly from a UK primary care medical records and therefore missing data are expected. There was no imputation in this study. For ethnicity and deprivation scores, missing data were categorized into a specific category to avoid creating bias in regression models.
  - Completeness of follow-up: this study used data from the IMRD database, with outcomes assessed until the earliest of the following time points: (1) recording of the outcome of interest; (2) patient censorship due to death or de-registration from their registered practice; (3) practice censorship due to ceasing of their data contribution to IMRD; and (4) study end date of 31 December 2020. Censorship happens if a patient de-registers from their general practice during the study or due to ceasing of their data contribution to IMRD.
  - Data linkage: there is no linkage to external data sources in this study.
- CODE-EHR Framework Domain 2: data fit for purpose
  - Origin, process and purpose of data: IMRD uses real-world data collected in primary care practices in the UK. Coded medical data will have been inputted by clinical or administrative staff during routine primary healthcare episodes or after correspondence from secondary care appointments/admissions. High-quality data are incentivized in the NHS through the Quality and Outcomes Framework, which measures practice performance and is used for billing and reimbursement purposes.
  - Coding systems: IMRD uses the read code coding system. Read codes are a systematic coding tool that have been used in the NHS since 1985. There were two versions in use during the study period, v.2 and CTV3 (v.3). Both versions provide a standard vocabulary for clinicians to record patient findings and management in health and social care systems across primary and secondary care.
  - Quality assessment: to ensure data quality in this study, practice data were only included if the practice had contributed to the IMRD database for at least 1 year and had mortality rates comparable to national averages. A further quality check was conducted in patient inclusion criteria, with patients required to have been registered at their general practice for at least 1 year during the time of the study.
  - Potential sources of bias: the IMRD database encompasses 6% of the population of the United Kingdom, with a slight skew toward areas with younger and more affluent patients. Despite this, the prevalence of diseases is consistent with wider UK data and is generalizable for demographics and major condition prevalence^38^. The NHS is a publicly funded healthcare system and individual clinicians do not receive personal funding for coded healthcare events.
- CODE-EHR Framework Domain 3: disease and outcome definitions
  - Definitions: code lists were developed for inclusion criteria, baseline characteristics and outcome events, and uploaded to a publicly accessible website (https://www.birmingham.ac.uk/research/cardiovascular-sciences/research/dare2think/connected-research/connected-research.aspx).
  - Coding manual: the DExtER tool was used to implement the curated code lists for direct data extraction of relevant patients. The coding manual was published online on 23/03/2023.
  - Phenotyping approaches: patients were included in the exposed cohort if there was a code for AF in their record. For the study exclusion criteria, a CHA_2_DS_2_-VASc score was calculated for each patient based on the code lists described. One point was given for a code relating to hypertension, diabetes, heart failure or vascular disease, and two points for stroke, transient ischemic attack or arterial thromboembolism.
  - Validation of coding: the code lists used in this study were developed using the DExtER tool after use and validation in previous studies. All code lists were examined and, where needed, updated before analysis. Outcome code lists benefited from a multi-stage validation process undertaken for the DaRe2THINK randomized controlled trial (fully described in the published method paper at 10.1093/ehjdh/ztac046)^13^.
- CODE-EHR Framework Domain 4: analysis
  - Statistical methods: please see Methods section.
  - Machine code: no machine code or algorithms were used in the analysis.
  - Internal validation: cross checks were made of crude unadjusted event rates, incident rates and adjusted data. Regression models were tested for model fit and proportionality and sensitivity analyses were performed as described in the main Methods section.
  - External validation: the data used in this study were pooled from 828 independent general practices, each with a varying number of General Practitioners (England average in 2022 of 5.5 full-time doctors per practice). No other data sources were used.
- CODE-EHR Framework Domain 5: ethics and governance
  - Consent: IMRD contains fully anonymized data extracted directly from general practice medical records. Data collection for IMRD was approved by the NHS South-East Multicentre Research Ethics Committee. NHS data in England are collected within an ‘opt-out’ approach, meaning that consent is not required. The study was conducted in accordance with the ethical principles set out in the Helsinki Declaration and Recommendations for Good Clinical Practice.
  - Data privacy: data are collected automatically from electronic health records from participating general practices. This includes information about patients’ health such as their diseases, test results and medication, but not their name, address or other information that could directly identify them. Patients who do not wish for their data to be used for research can opt out, through local and national data opt-out mechanisms.
  - Patient and public involvement: patients and the public were involved during all aspects of the study program, from inception to dissemination. Patient and public involvement was coordinated through the card*AI*c programme at the University of Birmingham/University Hospitals Birmingham NHS Foundation Trust (Clinical and Data Science Forum) and funded through the RATE-AF and DaRe2THINK trials (National Institute for Health and Care Research).
  - Data sharing: summary data are available upon reasonable request by contacting the corresponding author. The sharing of individual patient data from this study is not possible and would require further ethical approval.

### Reporting summary

Further information on research design is available in the Nature Portfolio Reporting Summary linked to this article.

## Online content

Any methods, additional references, Nature Portfolio reporting summaries, source data, extended data, supplementary information, acknowledgements, peer review information; details of author contributions and competing interests; and statements of data and code availability are available at 10.1038/s41591-024-03049-9.

### Supplementary information

Reporting Summary

## Figures and Tables

**Extended Data Table 1:**
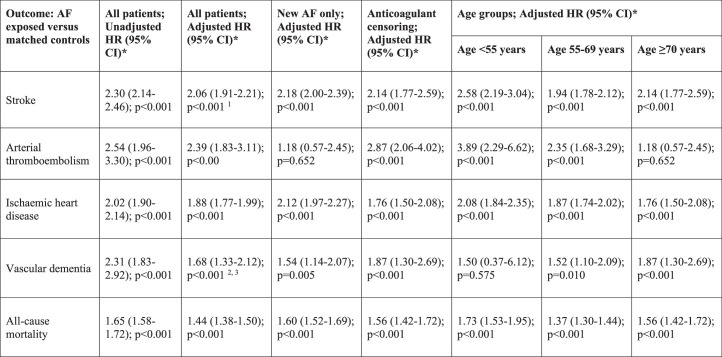
Subgroup and sensitivity analyses

All hazard ratios (HR) are for those exposed to atrial fibrillation (AF) compared to matched controls. * Using Cox proportional hazards regression models for each outcome (where adjusted, this accounts for age, sex, Townsend deprivation index, ethnicity, previous hypertension and diabetes mellitus), with a two-tailed p value. 1 Sub-distribution HR 2.00 after accounting for the competing risk of death (95% CI 1.85–2.15; p < 0.001). 2 Sub-distribution HR 1.59 after accounting for the competing risk of death (95% CI 1.26–2.01; p < 0.001). 3 Sub-distribution HR 2.14 after accounting for the competing risk of ischemic stroke or ischemic heart disease (95% CI 1.69–2.70; p < 0.001).

## Data Availability

Access to anonymized patient data from IMRD is subject to a data-sharing agreement and protocol approval from the IMRD Scientific Review Committee. The study-specific analyzable dataset is therefore not publicly available; however, this can be shared after obtaining approvals through contact with the corresponding author (D.K.; d.kotecha@bham.ac.uk; 60-day response time for decisions). Details about IMRD applications and data access are available on the IQVIA Medical Research Data website.

## Code availability

Code lists were developed for inclusion criteria, baseline characteristics and outcome events, and uploaded to a publicly accessible website before analysis (https://www.birmingham.ac.uk/research/cardiovascular-sciences/research/dare2think/connected-research/connected-research.aspx). Analysis scripts for this study are available from the corresponding author on request, after appropriate approvals from IMRD as noted in the data availability statement (D.K.; d.kotecha@bham.ac.uk; 60-day response time for decisions).
