# Supplementary material for: Thromboembolic events and vascular dementia in patients with atrial fibrillation and low apparent stroke risk
Source: Nat Med. 2024 Jun 5;30(8):2288–94. doi: 10.1038/s41591-024-03049-9 (PMC11333279; doi:10.1038/s41591-024-03049-9)
Supplement: Supplementary file 1 — Reporting Summary [file 41591_2024_3049_MOESM1_ESM.pdf]

nature portfolio

Corresponding author(s): Professor Dipak Kotecha

Last updated by author(s): Apr 26, 2024

Reporting Summary

Nature Portfolio wishes to improve the reproducibility of the work that we publish. This form provides structure for consistency and transparency in reporting. For further information on Nature Portfolio policies, see our Editorial Policies and the Editorial Policy Checklist.

Statistics

For all statistical analyses, confirm that the following items are present in the figure legend, table legend, main text, or Methods section.

n/a	Confirmed	
☐	☒	The exact sample size (n) for each experimental group/condition, given as a discrete number and unit of measurement
☒	☐	A statement on whether measurements were taken from distinct samples or whether the same sample was measured repeatedly
☐	☒	The statistical test(s) used AND whether they are one- or two-sided<br>*Only common tests should be described solely by name; describe more complex techniques in the Methods section.*
☐	☒	A description of all covariates tested
☐	☒	A description of any assumptions or corrections, such as tests of normality and adjustment for multiple comparisons
☐	☒	A full description of the statistical parameters including central tendency (e.g. means) or other basic estimates (e.g. regression coefficient) AND variation (e.g. standard deviation) or associated estimates of uncertainty (e.g. confidence intervals)
☐	☒	For null hypothesis testing, the test statistic (e.g. F, t, r) with confidence intervals, effect sizes, degrees of freedom and P value noted<br>*Give P values as exact values whenever suitable.*
☒	☐	For Bayesian analysis, information on the choice of priors and Markov chain Monte Carlo settings
☒	☐	For hierarchical and complex designs, identification of the appropriate level for tests and full reporting of outcomes
☒	☐	Estimates of effect sizes (e.g. Cohen's d, Pearson's r), indicating how they were calculated

Our web collection on statistics for biologists contains articles on many of the points above.

Software and code

Policy information about availability of computer code

Data collection	Data extraction for this study was conducted using the Data Extraction for Epidemiological Research (DExtER) tool: Gokhale, K.M., et al. Data extraction for epidemiological research (DExtER): a novel tool for automated clinical epidemiology studies. Eur J Epidemiol 36, 165-178 (2021).
Data analysis	All analysis were conducted in Stata (StataCorp, Texas)

For manuscripts utilizing custom algorithms or software that are central to the research but not yet described in published literature, software must be made available to editors and reviewers. We strongly encourage code deposition in a community repository (e.g. GitHub). See the Nature Portfolio guidelines for submitting code & software for further information.

Data

Policy information about availability of data

All manuscripts must include a data availability statement. This statement should provide the following information, where applicable:

- Accession codes, unique identifiers, or web links for publicly available datasets
- A description of any restrictions on data availability
- For clinical datasets or third party data, please ensure that the statement adheres to our policy

Access to anonymized patient data from IMRD is subject to a data sharing agreement and protocol approval from the IMRD Scientific Review Committee. The study-specific analysable dataset is therefore not publicly available, however can be shared after obtaining approvals through contact with the corresponding

author (Prof Dipak Kotecha; d.kotecha@bham.ac.uk; 60-days response time for decisions). Details about IMRD applications and data access are available on the IQVIA Medical Research Data website.

Research involving human participants, their data, or biological material

Policy information about studies with human participants or human data. See also policy information about sex, gender (identity/presentation), and sexual orientation and race, ethnicity and racism.

Reporting on sex and gender	This study collected the biological sex of all participants included in the study using the designated category in the participants medical record. A participants chosen gender was not taken into account due to a lack of this information in UK primary care records. Adjusted models of analysis adjusted for sex difference between the two comparator groups.
Reporting on race, ethnicity, or other socially relevant groupings	Information about a patients socioeconomic status and ethnicity as these have the potential to impact on the rates of outcomes accessed in this study. Socioeconomic status was recorded as the Townsend deprivation index categorized into quintiles. Ethnicity was categorised into: (1) White Caucasian; (2) Black Afro-Caribbean; (3) South Asian; (4) Mixed or multiple ethnicities; and (5) Other ethnic group. Patients with missing Townsend and ethnicity data were aggregated into a separate missing category within that variable. Both variables were included in all adjusted analysis for each study outcome.
Population characteristics	Overall median age of 58.2 years (IQR 51.2-63.1), 18.0% women, 2.1% with diabetes and 16.5% with hypertension. See Table 1 for further details.
Recruitment	Participants were included in this study if they were registered at an eligible general practice for at least a year during the study period and met the trials inclusion and exclusion criteria. IMRD database encompasses 6% of the population of the UK, with a slight skew towards areas with younger and more affluent patients. Despite this, the prevalence of diseases is consistent with wider UK data, and is generalisable for demographics and major condition prevalence.5 The NHS is a publicly-funded healthcare system and individual clinicians do not receive personal funding for coded healthcare events.
Ethics oversight	Data collection for IMRD was approved by the National Health Service South-East Multicentre Research Ethics Committee in 2003. Under the terms of this approval, each study protocol undergoes independent review from the Scientific Review Committee (approval July 2017; reference number SRC 17THIN061).

Note that full information on the approval of the study protocol must also be provided in the manuscript.

Field-specific reporting

Please select the one below that is the best fit for your research. If you are not sure, read the appropriate sections before making your selection.

☒ Life sciences ☐ Behavioural & social sciences ☐ Ecological, evolutionary & environmental sciences

For a reference copy of the document with all sections, see nature.com/documents/nr-reporting-summary-flat.pdf

Life sciences study design

All studies must disclose on these points even when the disclosure is negative.

Sample size	No sample size calculation performed.
Data exclusions	No data was excluded from the final analysis instead missing data was categorised into a specific category to avoid creating bias in regression models and censorship occurred due to death or de-registration from their registered practice or due to ceasing of practice data contribution to IMRD.
Replication	Attempts at replication were successful - see 'Subgroup and Sensitivity analyses' section in main results for details.
Randomization	Patients with a recorded diagnosis of AF were included in the exposed cohort these. For each exposed patient, up to 4 patients without an AF diagnosis were randomly selected after matching for age (±1 year), sex and the health authority region of the patient's registered general practice. The same index date of the exposed patient was assigned to the corresponding matched unexposed patients to avoid immortal time bias
Blinding	Blinding is not relevant possible for this population-based, retrospective, matched open cohort study.

Reporting for specific materials, systems and methods

We require information from authors about some types of materials, experimental systems and methods used in many studies. Here, indicate whether each material, system or method listed is relevant to your study. If you are not sure if a list item applies to your research, read the appropriate section before selecting a response.

Materials & experimental systems

n/a	Involved in the study
☒	☐ Antibodies
☒	☐ Eukaryotic cell lines
☒	☐ Palaeontology and archaeology
☒	☐ Animals and other organisms
☒	☐ Clinical data
☒	☐ Dual use research of concern
☒	☐ Plants

Methods

n/a	Involved in the study
☒	☐ ChIP-seq
☒	☐ Flow cytometry
☒	☐ MRI-based neuroimaging

Plants

Seed stocks	NA
Novel plant genotypes	NA
Authentication	NA
